# Hyperlipidemia and mortality in patients on peritoneal dialysis

**DOI:** 10.1186/s12882-022-02970-w

**Published:** 2022-10-24

**Authors:** Xiaoran Feng, Xiaojiang Zhan, Yueqiang Wen, FenFen Peng, Xiaoyang Wang, Niansong Wang, Xianfeng Wu, Junnan Wu

**Affiliations:** 1 Department of Nephrology, Jiujiang No. 1 People’s Hospital, Jiujiang, China; 2 Department of Nephrology, the First Affiliated Hospital of Nanchang University, Nanchang, China; 3 Department of Nephrology, the Second Affiliated Hospital of Guangzhou Medical University, Guangzhou, China; 4 Department of Nephrology, Zhujiang Hospital of Southern Medical University, Guangzhou, China; 5 Department of Nephrology, The First Affiliated Hospital of Zhengzhou University, Zhengzhou, China; 6 Department of Nephrology, Affiliated Sixth People’s Hospital, Shanghai Jiao Tong University, Shanghai, China; 7 Clinical Research Center for Chronic Kidney Disease, Shanghai Jiao Tong University Affiliated Sixth People’s Hospital, Shanghai, China; 8 Department of Nephrology, Zhejiang University Medical College Affiliated Sir Run Run Shaw Hospital, Qingchun Road 3rd, 310016 Hangzhou, Zhejiang Province China

**Keywords:** Peritoneal dialysis, Mortality, Hyperlipidemia, Hypertension

## Abstract

**Background:**

New lipid-lowering therapy at the start of dialysis and measurement of lipid parameters over the follow-up period is not recommended in dialysis patients, which seems unappropriated in clinical practice. We aimed to examine the effect of hyperlipidemia on mortality in patients undergoing continuous ambulatory peritoneal dialysis (CAPD).

**Methods:**

A retrospective cohort study was performed, including 2939 incident CAPD patients from five dialysis facilities between January 1, 2005, and December 31, 2018. The primary outcome was all-cause mortality. The association between hyperlipidemia at the start of CAPD and all-cause mortality was evaluated using Cox proportional hazards regression.

**Results:**

Of 2939 with a median age of 50.0 (interquartile range, 39.0–61.0), 1697 (57.7%) were men, 533 (18.1%) had hyperlipidemia, 549 (18.7%) had diabetes mellitus, 1915 (65.2%) had hypertension, and 410 (14.0%) had a history of CVD. During the median follow-up period of 35.1 months, 519 (17.7%) died, including 402 (16.7%, 47.4/1000 patient-years) in the non-hyperlipidemia group and 117 (22.0%, 71.1/1000 patient-years) in the hyperlipidemia group. Over the overall follow-up period, patients with hyperlipidemia had an equally high risk of all-cause mortality throughout follow-up as those without hyperlipidemia ([HR] 1.04, 95% confidence interval [CI] 0.83 to 1.31). However, from the 48-month follow-up onwards, hyperlipidemia was associated with a 2.26 (95% CI 1.49 to 3.43)-time higher risk of all-cause mortality than non-hyperlipidemia. Hypertension modified the association between hyperlipidemia and all-cause mortality (P for interaction < 0.001). A significantly increased risk of all-cause mortality was observed among patients with hypertension (HR 2.27, 95%CI 1.44–3.58).

**Conclusion:**

Among CAPD patients, hyperlipidemia at the beginning of CAPD was associated with a high risk of long-term mortality. Hypertension may mediate the association. Our findings suggested that long-term lipid-lowering treatment should be used in those patients with hyperlipidemia.

**Supplementary information:**

The online version contains supplementary material available at 10.1186/s12882-022-02970-w.

## Introduction

In the general population, a clear association exists between lipid profiles and major atherosclerotic events [1, 2]. In dialysis patients, lipid-lowering therapy has not a beneficial effect on patients’ survival [3–5]. Patients with end-stage renal disease have a characteristic lipid pattern of hypertriglyceridemia and low high-density cholesterol levels but normal low-density cholesterol levels [6]. Patients on peritoneal dialysis (PD) presented more markedly abnormal lipid profiles than those on hemodialysis, probably because of the metabolic influence of the peritoneal dialysis fluid [7–10]. The KDIGO guideline suggests that lipid-lowering therapy should be continued in patients already receiving the treatment at the dialysis initiation time but not be initiated in those not receiving the treatment [11]. These recommendations seem unappropriated in clinical practice. Although two studies did not examine the association between lipid-lowering therapy and mortality in dialysis patients with long-term follow-up, the survival plot showed that lipid-lowering treatment provided better survival in those with long-term follow-up. Therefore, we hypothesized that hyperlipidemia might harm long-term survival in peritoneal dialysis patients. Our study aimed to evaluate the association between hyperlipidemia at the start of PD and long-term all-cause mortality in patients on continuous ambulatory peritoneal dialysis (CAPD).

## Materials and methods

### Study design and population

A retrospective cohort study was performed, including 3073 incident CAPD patients from five peritoneal dialysis centers of three provinces in China between January 1, 2005, and December 31, 2018. To increase the generalizability of the findings in the CAPD settings, we only excluded those with age < 18 years or less than 3 months of follow-up. The Human Ethics Committee approved each research facility’s study, consistent with the Declaration of Helsinki’s ethical principles. Written informed consent was obtained from all eligible patients.

### Data collection and definitions

Two trained nurses in each dialysis center recorded demographic data, comorbidities, medications, and laboratory parameters at baseline. The baseline was defined as one month before the first CAPD. If the patient has more than one measurement in this one month, the measurement closest to the first CAPD will be included. If parameters were missed at baseline, parameters closest to before the first CAPD were included. Medications included calcium channel blockers, beta-blockers, angiotensin II receptor blockers/angiotensin-converting enzyme inhibitors (ACEI/ARBs), diuretics, statins, and aspirin. Baseline laboratory parameters included hemoglobin, serum albumin, serum uric acid, estimated glomerular filtration rate (eGFR), cholesterol, triglyceride, high-density lipoprotein, and low-density lipoprotein, and high-sensitivity C-reactive protein [hs-CRP]). All laboratory parameters from fasting blood samples were measured in the department of the laboratory of each tertiary hospital.

Hyperlipidemia was defined as serum cholesterol levels ≥ 240 mg/dL, triglycerides levels ≥ 200 mg/dL, low-density lipoprotein levels ≥ 160 mg/dL, or when the patients were receiving lipid-lowering drugs [12]. Patients with a history of CVD receiving lipid-lowering medications to prevent recurrence of CVD episodes were not considered hyperlipidemia. Diabetes mellitus was defined as ⑴ HbA1c ≥ 6.5%, ⑵ fasting plasma glucose ≥ 126 mg/dL, ⑶ 2-hour plasma glucose ≥ 200 mg/dL during an OGTT, ⑷ in a patient with classic symptoms of hyperglycemia or hyperglycemic crisis, a random plasma glucose ≥ 200 mg/dL, ⑸ or when the patients were receiving glucose-lowering drugs. In the absence of unequivocal hyperglycemia, criteria ⑴ to ⑶ should be confirmed by repeat testing.[13] Hypertension was defined as systolic blood pressure (BP) > 140 mmHg or diastolic BP > 90 mmHg or taking antihypertensive medications.[14] CVD was defined as coronary heart disease, congestive heart failure, arrhythmias, cerebrovascular disease, or peripheral vascular disease.[15] Current smoking was defined as at least one cigarette a day, and current alcohol consumption was defined as > 20 g of ethanol a day.[16] The comorbidity scores were calculated according to the Charlson comorbidity index calculator, which categorizes patients’ comorbidities. The more points are given, the more likely the predicted adverse outcomes are.[17] eGFR was calculated using the Chronic Kidney Disease Epidemiology Collaboration equation.[18].

### Outcomes and follow-Up

The primary outcome was all-cause mortality. Two experts identified the exact death cause based on death certificates and medical records. We determined death causes and dates based on medical files of admission. If patients died out of hospitals, we determined death causes according to interviewing family members by telephone to acknowledge death’s circumstances, combined with information from medical records of peritoneal dialysis centers.

According to the KDIGO guideline,[11] hyperlipidemia patients in our study not receiving lipid-lowering therapy did not receive a new lipid-lowering treatment when starting CAPD. Patients needed to return quarterly to each facility for an overall medical assessment for clinical purposes. The trained nurses conducted monthly face-to-face interviews or telephone interviews to assess the patient’s general conditions related to medications. If patients had significant serum lipid disorders during the follow-up period, we conducted an integrated approach rather than statins to improve serum lipid disorders[19]. All patients were followed up until CAPD cessation, death, the end of the 8-year duration, or June 30, 2019. Transferring to hemodialysis, receiving renal transplantation, moving to other centers, loss of follow-up, or still survival with a follow-up period of 8 years or as of June 30, 2019, were considered censored.

### Statistical analysis

We summarized baseline characteristics using descriptive statistics and expressed continuous variables as the mean (standard deviations) or median (interquartile range, [IQR]) and categorical variables as frequency (percentage). Multivariable binary logistic regression was conducted to estimate the association between baseline variables and hyperlipidemia. Covariables in multivariable binary logistic regression included age, sex, body mass index, systolic BP, diastolic BP, 24-hour urine volume, current smoking, current alcohol consumption, Charlson comorbidity index, diabetes mellitus, hypertension, and a history of CVD, hemoglobin, serum albumin, serum uric acid, eGFR, and hs-CRP.

Kaplan-Meier curve was conducted to examine the survival probability over the overall observational period. We constructed four Cox proportional hazards regression models to analyze the association between hyperlipidemia and all-cause mortality. Model 1, unadjusted; model 2, model 1 plus age, sex, body mass index, systolic BP, diastolic BP, current smoking, current alcohol consumption, 24-hour urine volume, Charlson comorbidity index, diabetes mellitus, hypertension, and a history of CVD; model 3, model 2 plus calcium channel blockers, beta-blockers, ACEI/ARBs, diuretics, statins, and aspirin; model 4, model 3 plus hemoglobin, serum albumin, serum uric acid, eGFR, cholesterol, triglyceride, high-density lipoprotein, low-density lipoprotein, and hs-CRP. Subgroups were stratified by age (< 65 or ≥ 65 years old), sex (men or women), diabetes mellitus (yes or no), hypertension (yes or no), and a history of CVD (yes or no). The interactions between subgroups and hyperlipidemia were examined by conducting a formal interaction test.

Many participants were censored due to hemodialysis transfer, kidney transplantations, moving to other centers, or loss of follow-up. Therefore, multiple subdistribution hazards models considering censoring events as competing risks were conducted as a sensitivity analysis. Additionally, the cumulative incidence was depicted using Gray’s test. We conducted a sensitivity analysis by excluding the patients already on lipid-lowering therapies from the whole cohort. The Cox proportional and subdistribution hazardresults were presented as the hazard ratio (HR) and the 95% confidence interval (CI). A two-sided *P* value < 0.05 was considered statistically significant. Statistical analyses were performed using Stata 15.1. statistical software (StataCorp, College Station, TX).

## Results

### Baseline characteristics

Of potential 3073 patients, 42 aged < 18 years and 92 with < 3-month dialysis duration were excluded. Finally, the remaining 2939 patients were eligible for analysis. Of 2939 with a median age of 50.0 (IQR, 39.0–61.0), 1697 (57.7%) were men, 533 (18.1%) had hyperlipidemia, 549 (18.7%) had diabetes mellitus, 1915 (65.2%) had hypertension, and 410 (14.0%) had a history of CVD. Table 1 showed variables at baseline stratified by hyperlipidemia over the overall follow-up period and 48-month follow-up onwards. Over the follow-up period, hyperlipidemia patients were more likely to be elderly, have current smoking, diabetes mellitus, hypertension, a history of CVD, take medications (except diuretics), and have a higher Charlson comorbidity index and hemoglobin compared with those without. From 48-month follow up onwards, patients with hyperlipidemia were more likely to be elderly, women, current smoking, current alcohol consumption, diabetes mellitus, hypertension, a history of CVD, taking medications (except diuretics), had higher levels of Charlson comorbidity index, hemoglobin, and cholesterol, compared to those without.

**Table 1:** Baseline characteristics and variables stratified by hyperlipidemia

	Over the overall follow-up period			From 48-month follow up onwards		
	Non-hyperlipidemia	Hyperlipidemia	***P***-value	Non-hyperlipidemia	Hyperlipidemia	***P***-value
N	2406	533		912	158	
Age, years	49.0 (38.0–60.0)	54.0 (43.0–64.0)	< 0.001	46.0 (37.0–57.0)	52.0 (42.2–63.0)	< 0.001
Men, %	1423 (59.1%)	274 (51.4%)	0.001	544 (59.6%)	79 (50.0%)	0.023
Body mass index, kg/m^2^	22.1 ± 6.3	23.0 ± 3.8	0.113	22.1 ± 6.3	23.0 ± 3.8	0.113
Systolic BP, mmHg	140.8 ± 26.2	141.9 ± 25.2	0.652	140.8 ± 26.2	141.9 ± 25.2	0.652
Diastolic BP, mmHg	89.5 ± 16.7	87.0 ± 15.4	0.088	89.5 ± 16.7	87.0 ± 15.4	0.088
24-hour urine volume, mL	800 (450–1200)	825 (500–1288)	0.249	800 (450–1200)	825 (500–1288)	0.249
Current smoking, (%)	228 (9.5%)	66 (12.4%)	0.043	60 (6.6%)	21 (13.3%)	0.003
Current alcohol consumption, (%)	82 (3.4%)	26 (4.9%)	0.103	18 (2.0%)	9 (5.7%)	0.006
Charlson comorbidity index	3.3 ± 0.6	3.6 ± 0.8	< 0.001	3.2 ± 0.5	3.5 ± 0.7	< 0.001
Diabetes mellitus, (%)	371 (15.4%)	178 (33.4%)	< 0.001	97 (10.6%)	42 (26.6%)	< 0.001
Hypertension, (%)	1527 (63.5%)	388 (72.8%)	< 0.001	538 (59.0%)	110 (69.6%)	0.012
A history of CVD, (%)	274 (11.4%)	136 (25.5%)	< 0.001	94 (10.3%)	26 (16.5%)	0.024
Calcium channel blockers, (%)	1496 (62.2%)	358 (67.2%)	< 0.001	502 (55.0%)	98 (62.0%)	< 0.001
Beta blockers, (%)	923 (38.4%)	290 (54.4%)	< 0.001	388 (42.5%)	85 (53.8%)	0.009
Diuretics, (%)	181 (7.5%)	19 (3.6%)	0.001	43 (4.7%)	7 (4.4%)	0.876
ACEI/ARBs, (%)	760 (31.6%)	252 (47.3%)	< 0.001	329 (36.1%)	83 (52.5%)	< 0.001
Aspirin, (%)	183 (7.6%)	61 (11.4%)	0.004	58 (6.4%)	33 (20.9%)	< 0.001
Statins, (%)	269 (11.2%)	147 (27.6%)	< 0.001	58 (6.4%)	26 (16.5%)	< 0.001
Hemoglobin, g/dL	9.1 (2.8)	10.0 (2.7)	< 0.001	9.2 ± 3.0	10.1 ± 2.5	< 0.001
Serum albumin, g/dL	3.5 (0.6)	3.4 (0.5)	0.449	3.5 ± 0.6	3.4 ± 0.5	0.587
Serum uric acid, mg/dL	6.9 (2.4)	6.9 (2.3)	0.696	6.9 ± 2.5	7.1 ± 2.2	0.325
eGFR, mL/min/1.73 m^2^	6.5 (4.7–8.3)	6.4 (4.7–8.3)	0.976	6.5 (4.7–8.3)	6.3 (4.4–8.2)	0.208
Cholesterol, mg/dL	151.2 (117.2–182.0)	154.7 (121.4-185.6)	0.117	149.3 (116.4-183.1)	161.8 (129.2-189.9)	0.023
Triglyceride, mg/dL	94.8 (56.7-153.3)	92.1 (55.8-151.6)	0.753	94.8 (57.4-154.2)	87.7 (53.2-152.6)	0.720
High-density lipoprotein, mg/dL	39.4 (31.3–49.9)	39.6 (31.8–49.1)	0.939	39.4 (29.8–48.8)	38.3 (32.7–49.5)	0.611
Low-density lipoprotein, mg/dL	81.3 (47.4-116.3)	83.9 (48.7-119.9)	0.127	80.4 (49.8-117.5)	83.3 (41.0-121.0)	0.527
hs-CRP, mg/L	4.3 (1.9–14.0)	4.5 (1.9–16.0)	0.622	4.2 (1.9–13.2)	4.9 (2.0-18.5)	0.065

BP, blood pressure; CVD, cardiovascular disease; ACEI/ARBs, angiotensin II receptor blockers/angiotensin-converting enzyme inhibitors; eGFR, estimated glomerular filtration rate; hs-CRP, high-sensitivity C-reactive protein

### Association between baseline parameters and hyperlipidemia

Table 2 showed that over the overall follow-up period, women, current smoking, higher levels of Charlson comorbidity index, body mass index, and hemoglobin were independently associated with hyperlipidemia after adjusting for confounding factors. From the 48-month follow-up onwards, elderly age, women, current smoking, diabetes mellitus, and higher hemoglobin levels were independently associated with hyperlipidemia after adjusting for confounding factors.

**Table 2:** Independent risk factors for hyperlipidemia using multivariable binary logistic regression

Over the overall follow-up period	OR	95% CI	From 48-month follow up onwards	OR	95% CI
Women, men as a reference	1.63	1.33 to 2.00	Age, per increase 10 years	1.17	1.03 to 1.34
Charlson comorbidity index^#^	1.95	1.71 to 2.22	Women, men as a reference	1.96	1.34 to 2.86
Body mass index^&^	1.004	1.001 to 1.008	Current smoking, yes/no	2.82	1.57 to 5.08
Current smoking, yes/no	1.53	1.11 to 2.11	Diabetes mellitus, yes/no	2.78	1.79 to 4.32
Hemoglobin, per increase 1 g/dL	1.09	1.03 to 1.34	Hemoglobin, per increase 1 g/dL	1.09	1.03 to 1.34

^#^ per increase 1 score; ^&^ per increase 1 kg/m^2^

Covariables included age, sex, body mass index, systolic BP, diastolic BP, current smoking, current alcohol consumption, 24-hour urine volume, Charlson comorbidity index, diabetes mellitus, hypertension, a history of CVD, hemoglobin, serum albumin, serum uric acid, eGFR, and hs-CRP.

BP, blood pressure; CVD, cardiovascular disease; eGFR, estimated glomerular filtration rate; hs-CRP, high-sensitivity C-reactive protein; OR, odds ratio; CI, confidence interval

### Observational period and all-cause mortality

The observational period was 10122.2 patient years, with a median of 35.1 (IQR 17.9–61.7) months. During this period, 519 (17.7%) died, with 258 (8.8%) CVD deaths, 54 (1.8%) infection deaths, 9 (0.3%) gastrointestinal bleeding deaths, 16 (0.5%) tumor deaths, 93 (3.2%) other death causes, and 82 (2.8%) unknown death causes. By the end of the follow-up, 353 (12.0%) transferred to hemodialysis, 153 (5.2%) received renal transplants, 26 (0.9%) transferred to other dialysis centers, and 100 (3.4%) lost of follow-up.

Over the overall follow-up period, 402 (16.7%, 47.4/1000 patient-years) died in the non-hyperlipidemia group and 117 (22.0%, 71.1/1000 patient-years) in the hyperlipidemia group. 213 (8.9%) CVD deaths occurred in the non-hyperlipidemia group and 45 (8.4%) in the hyperlipidemia group. From the 48-month follow-up onwards, 77 (8.4%, 13.8/1000 patient-years) died in the non-hyperlipidemia group and 34 (21.5%, 36.0/1000 patient-years) in the hyperlipidemia group. In addition, from the 48-month follow-up onwards, 45 CVD deaths (4.9%) occurred in the non-hyperlipidemia group and 14 (8.9%) in the hyperlipidemia group.

### Hyperlipidemia and all-cause mortality

**Fig. 1:**
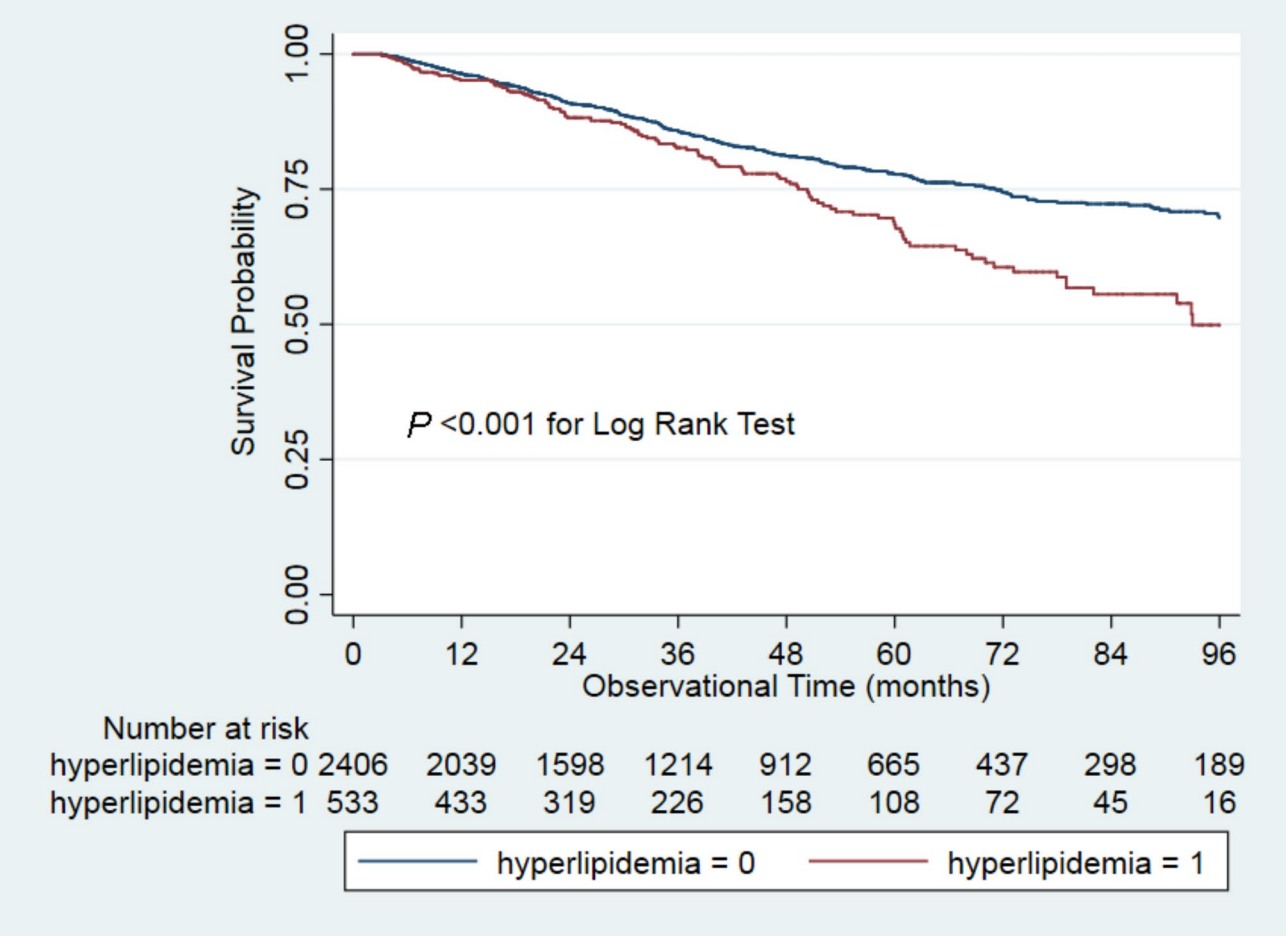
Survival probability between hyperlipidemia and non-hyperlipidemia groups

Survival analysis showed that survival probability significantly differed between hyperlipidemia and non-hyperlipidemia groups over the overall follow-up period (*P* < 0.001, Fig. 1). Notably, from the 48-month follow-up onwards, survival probability significantly differed between groups.

Table 3 shows that patients with hyperlipidemia had an equally high risk of all-cause mortality throughout follow-up as patients without hyperlipidemia (HR 1.04, 95% CI 0.83 to 1.31) in Model 4. From the 48-month follow-up onwards, patients with hyperlipidemia had a 2.26 (95% CI 1.49 to 3.43)-time higher risk of all-cause mortality than those without in model 4.

**Table 3:** Association between hyperlipidemia and all-cause mortality using Cox proportional hazards regression

	Model 1		Model 2		Model 3		Model 4	
	HR	95%CI	HR	95%CI	HR	95%CI	HR	95%CI
Over the overall follow-up period								
Hyperlipidemia (yes/no)	1.52	1.23 to 1.86	1.02	0.82 to 1.26	1.03	0.83 to 1.29	1.04	0.83 to 1.31
From 48-month follow-up onwards								
Hyperlipidemia (yes/no)	2.76	1.85 to 4.14	2.11	1.40 to 3.18	2.11	1.40 to 3.18	2.26	1.49 to 3.43

Model 1, unadjusted; model 2, model 1 plus age, sex, body mass index, systolic BP, diastolic BP, current smoking, current alcohol consumption, 24-hour urine volume, Charlson comorbidity index, diabetes mellitus, hypertension, and a history of CVD; model 3, model 2 plus calcium channel blockers, beta-blockers, ACEI/ARBs, diuretics, statins, and aspirin; model 4, model 3 plus hemoglobin, serum albumin, serum uric acid, eGFR, cholesterol, triglyceride, high-density lipoprotein, low-density lipoprotein, and hs-CRP.

BP, blood pressure; CVD, cardiovascular disease; ACEI/ARBs, angiotensin II receptor blockers/angiotensin-converting enzyme inhibitors; eGFR, estimated glomerular filtration rate; hs-CRP, high-sensitivity C-reactive protein; HR, hazard ratio; CI, confidence interval

### Subgroup analyses

**Fig. 2:**
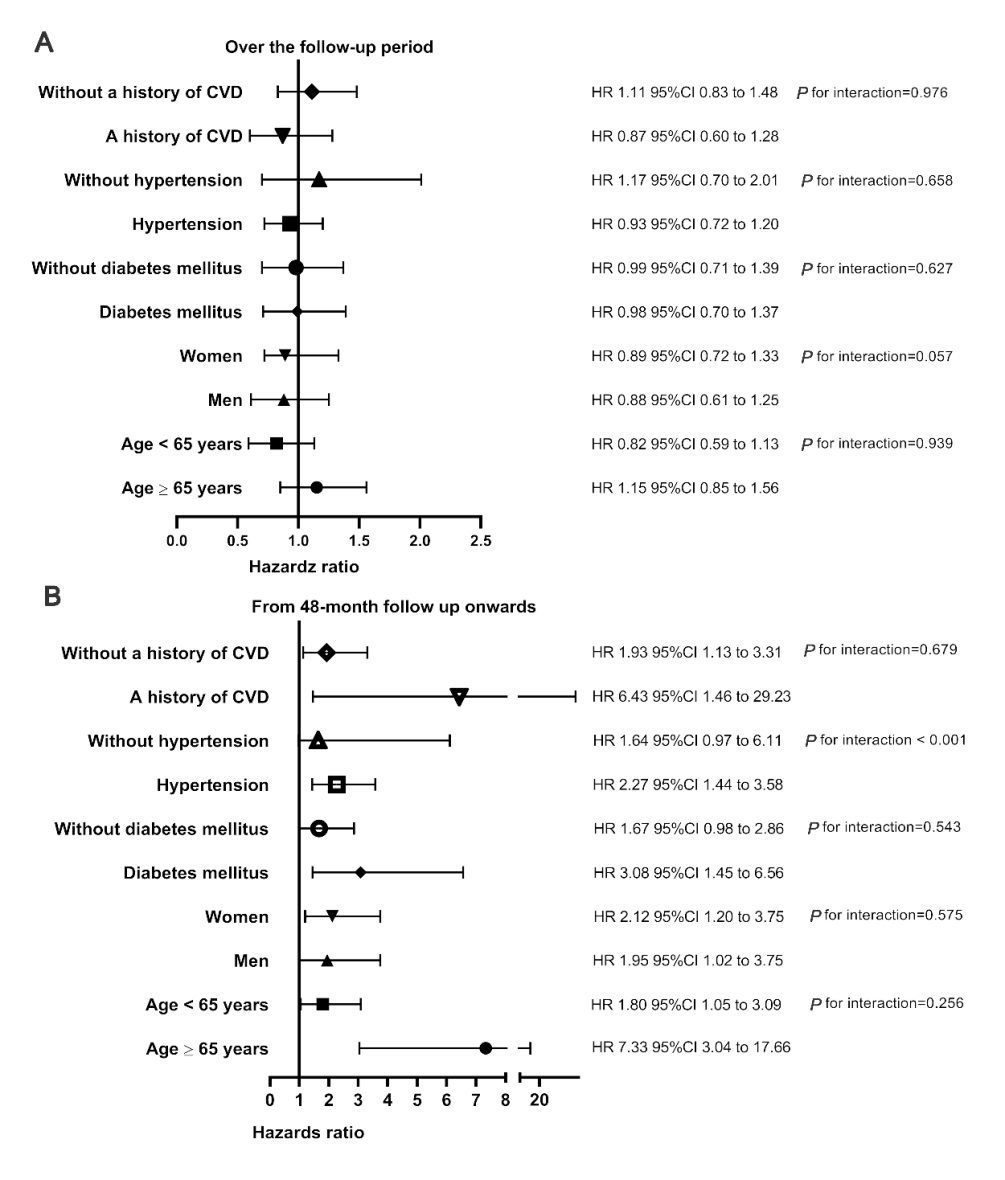
**Associations between hyperlipidemia and all-cause mortality in subgroups over the overall follow-up period (A) and from 48-month follow-up onwards (B)** Adjusted for age, sex, body mass index, systolic BP, diastolic BP, 24-hour urine volume, current smoking, current alcohol consumption, Charlson comorbidity index, diabetes mellitus, hypertension, and a history of CVD, calcium channel blockers, beta-blockers, ACEI/ARBs, diuretics, statins, aspirin, hemoglobin, serum albumin, serum uric acid, eGFR, cholesterol, triglyceride, high-density lipoprotein, low-density lipoprotein, and hs-CRP. BP, blood pressure; CVD, cardiovascular disease; ACEI/ARBs, angiotensin II receptor blockers/angiotensin-converting enzyme inhibitors; eGFR, estimated glomerular filtration rate; hs-CRP, high-sensitivity C-reactive protein; HR, hazards ratio; CI, confidence interval

To evaluate the modification effects of subgroups on the association between hyperlipidemia and all-cause mortality, we conducted subgroup analyses over the overall follow-up period and from the 48-month follow-up onwards. Over the overall follow-up period, hyperlipidemia did not affect all-cause mortality in all subgroups (Fig. 2 A). From the 48-month follow-up onwards, we found that hypertension modified the association between hyperlipidemia and all-cause mortality (P for interaction < 0.001, Fig. 2B). In further analysis, a significantly increased risk of all-cause mortality was observed among patients with hypertension (HR 2.27, 95%CI 1.44–3.58). In contrast, there was no significant association among those without hypertension. There were no other considerable subgroup interactions.

### Sensitivity analyses

**Fig. 3:**
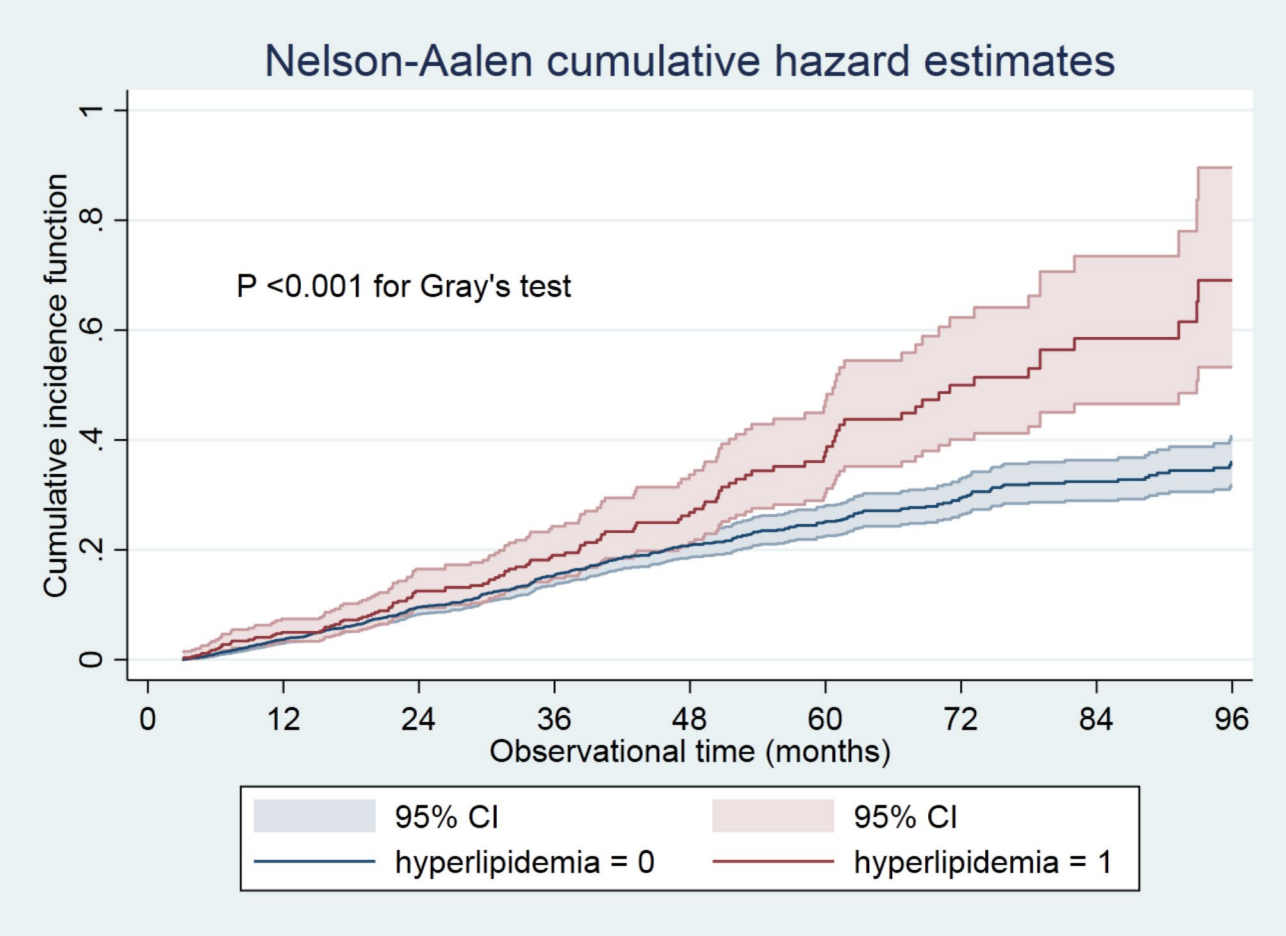
Cumulative incidence function between hyperlipidemia and non-hyperlipidemia groups

Cumulative incidence function in hyperlipidemia patients was significantly higher than in those without hyperlipidemia (Fig. 3). Similar trends were observed when evaluating the association using subdistribution hazard models (Table 4). Patients with hyperlipidemia had an equally high risk of all-cause mortality throughout follow-up as patients without hyperlipidemia (HR 1.16, 95% CI 0.94 to 1.43). From the 48-month follow-up onwards, patients with hyperlipidemia had a 2.15 (95% CI 1.42 to 3.25)-time higher risk of all-cause mortality than those without hyperlipidemia. We found a similar association when excluding the patients already on lipid-lowering therapies from the whole cohort. Patients with hyperlipidemia had an equally high risk of all-cause mortality throughout follow-up as patients without hyperlipidemia (HR 1.12, 95% CI 0.91 to 1.56). From the 48-month follow-up onwards, patients with hyperlipidemia had a 2.24 (95% CI 1.43 to 3.50)-time higher risk of all-cause mortality than those without hyperlipidemia.

**Table 4:** Association between hyperlipidemia and all-cause mortality using subdistribution hazards model

	Model 1		Model 2		Model 3		Model 4	
	HR	95%CI	HR	95%CI	HR	95%CI	HR	95%CI
Over the overall follow-up period								
Hyperlipidemia (yes/no)	1.49	1.22 to 1.82	1.14	0.93 to 1.39	1.13	0.92 to 1.38	1.16	0.94 to 1.43
From 48-month follow-up onwards								
Hyperlipidemia (yes/no)	2.85	1.90 to 4.26	2.22	1.48 to 3.31	2.12	1.42 to 3.18	2.15	1.42 to 3.25

Model 1, unadjusted; model 2, model 1 plus age, sex, body mass index, systolic BP, diastolic BP, current smoking, current alcohol consumption, 24-hour urine volume, Charlson comorbidity index, diabetes mellitus, hypertension, and a history of CVD; model 3, model 2 plus calcium channel blockers, beta-blockers, ACEI/ARBs, diuretics, statins, and aspirin; model 4, model 3 plus hemoglobin, serum albumin, serum uric acid, eGFR, cholesterol, triglyceride, high-density lipoprotein, low-density lipoprotein, and hs-CRP.

BP, blood pressure; CVD, cardiovascular disease; ACEI/ARBs, angiotensin II receptor blockers/angiotensin-converting enzyme inhibitors; eGFR, estimated glomerular filtration rate; hs-CRP, high-sensitivity C-reactive protein; HR, hazard ratio; CI, confidence interval

## Discussion

In this study, we found that over the overall follow-up period, hyperlipidemia was not associated with all-cause mortality, but from the 48-month follow-up onwards, hyperlipidemia was independently associated with an increased risk of all-mortality in CAPD patients. Notably, hypertension may mediate or confound the association in those with long-term follow-up periods. Our findings suggested that CAPD patients with hypertension should receive lipid-lowering therapy after the start of dialysis. Because hypertension is prevalent in dialysis patients, our results may be significant in clinical practice.

Three large clinical trials showed no benefit of statin therapy in dialysis patients [3–5]. The 2013 KDIGO for lipid management in chronic kidney disease has caused extensive discussion and controversy [20–22]. Notably, the KDIGO guideline recommends that lipid-lowering therapy not be initiated in dialysis patients not receiving the treatment, and levels of lipid parameters for most dialysis patients are not measured over the follow-up period [11]. We wondered about the association between hyperlipidemia and long-term survival in dialysis patients based on these backgrounds. In the present study, over the overall follow-up period, patients with hyperlipidemia were at as a high risk of all-cause mortality as those without hyperlipidemia, but from 48-month follow up onwards, hyperlipidemia patients had 2.26 (95% CI 1.49 to 3.43)-time higher risk of all-cause mortality than those without hyperlipidemia. Because two well-designed studies did not examine the association of lipid-lowering therapy with mortality in dialysis patients with a long-term follow-up period, we further observed survival trends in survival plots in those patients in the two studies. A study showed that from approximately 42-month follow-up onwards, diabetes mellitus patients with lipid-lowering therapy underdoing hemodialysis seemed to have better survival than those without lipid-lowering treatment [3]. Another study also reported that from approximately 50-month follow-up onwards of survival analysis, hemodialysis patients with lipid-lowering therapy seemed to have better survival than those without lipid-lowering treatment [4]. These findings suggested that hyperlipidemia at the start of dialysis therapy might harm long-term survival in CAPD patients. A large prospective study should verify our results.

In our study, it was worth noting that ⑴, in the study population, there was no significant difference in lipid profiles between patients with hyperlipidemia and those without hyperlipidemia. One reason behind the paradoxical finding may be that percentiles of taking statins in hyperlipidemia patients were significantly higher than those in those without hyperlipidemia (27.6% vs. 11.2%), which may lower the levels of lipid parameters such as cholesterol, triglyceride, high-density lipoprotein, and low-density lipoprotein in hyperlipidemia patients. According to the diagnosis of hyperlipidemia in the present study, meeting one criterion was defined as hyperlipidemia. Therefore, another reason may be that one patient with high cholesterol may have low triglycerides or low-density lipoprotein and vice versa. ⑵, Of 2406 patients without hyperlipidemia, 269 (11.2%) patients had received lipid-lowering therapy before starting CAPD. The reason for receiving lipid-lowering treatment in non-hyperlipidemia patients was to undergo the secondary prevention of CVD events in 274 (11.4%) those with a history of CVD.

The subgroup analyses highlighted the consistency in the association between hyperlipidemia and the risk of all-cause mortality across the younger and older, men and women, diabetes and non-diabetes, hypertension and non-hypertension, and a history of CVD and without a history of CVD. Over the overall follow-up period, hyperlipidemia was not associated with all-cause mortality in all subgroups. From the 48-month follow-up onwards, hyperlipidemia was not associated with all-cause mortality in all subgroups, except those with hypertension. These findings suggested that hypertension may mediate the association in those with long-term follow-up periods. Thus, future studies are warranted to examine the joint association of combining hyperlipidemia and hypertension with mortality in dialysis patients.

Several limitations should be discussed. First, this was a retrospective study with potential unaccounted confounding factors. Although confounding variables were adjusted, it cannot eliminate the residual confounding or reverse causality of hyperlipidemia and mortality. Second, although we had a detailed assessment of death, 82 (15.8%) deaths remained with unknown reasons, which may affect the association between hyperlipidemia and CVD mortality. We, therefore, did not evaluate the association between hyperlipidemia and CVD mortality. Third, although the KDIGO guideline does not recommend follow-up measurement of lipid parameters for most dialysis patients [23], we still at least quarterly measured these parameters for all CAPD patients. If patients had significant serum lipid disorders during the follow-up period, we performed an integrated approach, including lifestyle modifications, nutritional supplements, anti-inflammatory drugs, and improved dialysis therapy to improve serum lipid disorders.[19] However, given the influence of ethnic and regional characteristics on the Chinese CAPD population setting, the diagnosis of hyperlipidemia was based on the 2016 guidelines for managing hyperlipidemia in Chinese adults, which only included the hyperlipidemic population, not the hypolipidemic population [12]. This definition may lead to patients with lower lipid profiles being assigned to the non-hyperlipidemia group. Previous studies have reported lower lipid profiles are associated with coexisting malnutrition, inflammation, and increased mortality in dialysis patients [24–26]. Therefore, all-cause mortality in the non-hyperlipidemia may be over-estimated, which may more strongly support the association that hyperlipidemia harmed long-term all-cause mortality. Nonetheless, we did not evaluate the longitude effect of serum lipid parameters on mortality. Fourth, although we only focused on the effect of lipid profile before the first CAPD procedure on patient prognosis, the adequacy of dialysis during the follow-up period is very important to further understand our findings. Insufficient ultrafiltration can aggravate hypertension, but it can also require solutions of higher glucose concentration and modify the lipid profile, as well as require transfer to hemodialysis or lead to an earlier death. Thus, further studies should consider the effect of the adequacy of dialysis on the association between lipid profile and patient prognosis. Lastly, to enhance the generalizability of our findings in CAPD population settings, we only excluded those with age < 18 years or < 3-month period of follow-up. Nonetheless, all eligible patients were from China, suggesting our findings may lack generalization to other ethnic CAPD population settings.

In conclusion, hyperlipidemia at the start of CAPD was associated with an increased risk of long-term all-cause mortality in CAPD patients with long-term follow-up, and hypertension may mediate the association. Because hypertension is very prevalent in dialysis patients, our findings may be significant in clinical practice. Based on our findings, lipid-lowering treatment should be used in CAPD patients with hyperlipidemia. Future studies are warranted to evaluate the effect of lipid features on long-term survival in CAPD patients.

## Electronic supplementary material

Below is the link to the electronic supplementary material.

Supplementary Material 1

## Data Availability

The datasets used and/or analyzed during the current study are available from the corresponding author on reasonable request.
