# Supplementary material for: Hyperlipidemia and mortality in patients on peritoneal dialysis
Source: BMC Nephrol. 2022 Oct 24;23:342. doi: 10.1186/s12882-022-02970-w (PMC9590170; doi:10.1186/s12882-022-02970-w)
Supplement: Supplementary file 1 — Supplementary Material 1 [file 12882_2022_2970_MOESM1_ESM.doc]

**STROBE Statement**—checklist of items that should be included in reports of observational studies.

Hyperlipidemia and Mortality in Patients on Peritoneal Dialysis; Corresponding Author: Xianfeng Wu

|  | Item No | | Recommendation | Author’s Response |
| --- | --- | --- | --- | --- |
| **Title and abstract** | 1 | | (*a*) Indicate the study’s design with a commonly used term in the title or the abstract | See abstract. |
|  |  |  | (*b*) Provide in the abstract an informative and balanced summary of what was done and what was found | See “Background” and “Methods and Conclusions” in the Abstract. |
| Introduction | | | |  |
| Background/rationale | 2 | | Explain the scientific background and rationale for the investigation being reported | See Introduction. |
| Objectives | 3 | | State specific objectives, including any prespecified hypotheses | See Introduction. |
| Methods | | | |  |
| Study design | 4 | | Present key elements of study design early in the paper | See “Study Design and Population” in Methods. |
| Setting | 5 | | Describe the setting, locations, and relevant dates, including periods of recruitment, exposure, follow-up, and data collection | See “Study Design and Popuoation”, “Data Collection and Definitions”, and “Outcomes and Follow-up” in Methods. |
| Participants | 6 | | (*a*) *Cohort study*—Give the eligibility criteria, and the sources and methods of selection of participants. Describe methods of follow-up  *Case-control study*—Give the eligibility criteria, and the sources and methods of case ascertainment and control selection. Give the rationale for the choice of cases and controls  *Cross-sectional study*—Give the eligibility criteria, and the sources and methods of selection of participants | See “Study Design and Population”, and “Outcomes and Follow-up” in Methods. |
|  |  |  | (*b*) *Cohort study*—For matched studies, give matching criteria and number of exposed and unexposed  *Case-control study*—For matched studies, give matching criteria and the number of controls per case |  |
| Variables | 7 | | Clearly define all outcomes, exposures, predictors, potential confounders, and effect modifiers. Give diagnostic criteria, if applicable | See “Data Collection and Definitions”, and “Outcomes and Follow-up” in Methods. |
| Data sources/ measurement | 8 | | For each variable of interest, give sources of data and details of methods of assessment (measurement). Describe comparability of assessment methods if there is more than one group | See “Data Collection and Definitions”, in Methods. |
| Bias | 9 | | Describe any efforts to address potential sources of bias | See paragraph 2 in “Statistical analyses” in Methods. |
| Study size | 10 | | Explain how the study size was arrived at | See “Study Design and Population” in Methods. |
| Quantitative variables | 11 | | Explain how quantitative variables were handled in the analyses. If applicable, describe which groupings were chosen and why | See paragraph 1-2 in “Statistical analyses” in Methods |
| Statistical methods | 12 | | (*a*) Describe all statistical methods, including those used to control for confounding | See paragraph 1-2 in “Statistical analyses” in Methods. |
|  |  |  | (*b*) Describe any methods used to examine subgroups and interactions | See paragraph 2 in “Statistical analyses” in Methods |
|  |  |  | (*c*) Explain how missing data were addressed |  |
|  |  |  | (*d*) *Cohort study*—If applicable, explain how loss to follow-up was addressed  *Case-control study*—If applicable, explain how matching of cases and controls was addressed  *Cross-sectional study*—If applicable, describe analytical methods taking account of sampling strategy | See “Outcomes and Follow-up” in Methods. |
|  |  |  | (*e*) Describe any sensitivity analyses |  |
| Results | | | |  |
| Participants | 13 | (a) Report numbers of individuals at each stage of study—eg numbers potentially eligible, examined for eligibility, confirmed eligible, included in the study, completing follow-up, and analysed | | See “Baseline Characteristics” in Results. |
|  |  | (b) Give reasons for non-participation at each stage | |  |
|  |  | (c) Consider use of a flow diagram | |  |
| Descriptive data | 14 | (a) Give characteristics of study participants (eg demographic, clinical, social) and information on exposures and potential confounders | | See Table 1. |
|  |  | (b) Indicate number of participants with missing data for each variable of interest | | See “Data Collection and Definitions” in Methods.. |
|  |  | (c) *Cohort study*—Summarise follow-up time (eg, average and total amount) | | See “Observational Period and All-cause Mortality” in Results. |
| Outcome data | 15 | *Cohort study*—Report numbers of outcome events or summary measures over time | | See “Observational Period and All-cause Mortality” in Results. |
|  |  | *Case-control study—*Report numbers in each exposure category, or summary measures of exposure | |  |
|  |  | *Cross-sectional study—*Report numbers of outcome events or summary measures | |  |
| Main results | 16 | (*a*) Give unadjusted estimates and, if applicable, confounder-adjusted estimates and their precision (eg, 95% confidence interval). Make clear which confounders were adjusted for and why they were included | | See Results, Tables 3 and Fig 2. |
|  |  | (*b*) Report category boundaries when continuous variables were categorized | |  |
|  |  | (*c*) If relevant, consider translating estimates of relative risk into absolute risk for a meaningful time period | |  |
| Other analyses | 17 | Report other analyses done—eg analyses of subgroups and interactions, and sensitivity analyses | | See “sensitivity analyses”, and “subgroup analyses” in Results |
| Discussion | | | |  |
| Key results | 18 | Summarise key results with reference to study objectives | | See paragraph 1 and 6 in Discussion. |
| Limitations | 19 | Discuss limitations of the study, taking into account sources of potential bias or imprecision. Discuss both direction and magnitude of any potential bias | | See paragraph 5 in Discussion. |
| Interpretation | 20 | Give a cautious overall interpretation of results considering objectives, limitations, multiplicity of analyses, results from similar studies, and other relevant evidence | | See Discussion. |
| Generalisability | 21 | Discuss the generalisability (external validity) of the study results | | See paragraph 5 in Discussion. |
| Other information | | | |  |
| Funding | 22 | Give the source of funding and the role of the funders for the present study and, if applicable, for the original study on which the present article is based | | See information in the “Funding” field in the submission form. |
